# Editorial: Neuronal network dysfunction in neurodegenerative disorders

**DOI:** 10.3389/fnins.2023.1151156

**Published:** 2023-02-24

**Authors:** Andrea Kwakowsky, Asheeta A. Prasad, Fernando Peña-Ortega, Sean Austin Ong Lim

**Affiliations:** ^1^Pharmacology and Therapeutics, School of Medicine, Galway Neuroscience Centre, Ollscoil na Gaillimhe - University of Galway, Galway, Ireland; ^2^Department of Anatomy and Medical Imaging, Centre for Brain Research, Faculty of Medical and Health Science, University of Auckland, Auckland, New Zealand; ^3^Faculty of Medicine and Health, School of Medical Sciences, University of Sydney, Sydney, NSW, Australia; ^4^Departamento de Neurobiología del Desarrollo y Neurofisiología, Instituto de Neurobiología, Universidad Nacional Autónoma de México, Querétaro, Mexico; ^5^Neuroscience Program, College of Science and Health, DePaul University, Chicago, IL, United States

**Keywords:** neurodegeneration, neurotransmitter, neurodegenerative diseases, neuronal network remodeling, neuronal network dysfunction

The prevalence of neurodegenerative diseases is predicted to increase rapidly in the coming decades. There is a great need for therapies to prevent and/or slow the progression of these disorders as current therapies for most neurodegenerative diseases are symptomatic only. Neurodegenerative diseases such as Alzheimer's, Parkinson's, and Huntington's diseases as well as amyotrophic lateral sclerosis pose extraordinary challenges for drug development (Revi, 2020; Ferguson et al., 2022; Mead et al., 2022; Chopade et al., 2023). Several factors likely contribute to neurodegeneration, including oxidative stress, excitotoxicity, protein aggregation, vascular dysfunction, and neuroinflammation, and these processes culminate in the death of specific neuronal populations, leading to cognitive and/or motor impairments. The pathogenesis of neurodegenerative diseases is associated with significant dysfunction in multiple neurotransmitter systems, including altered levels of these neurotransmitters and the massive degeneration and remodeling of neuronal networks (Palop et al., 2006; Brichta et al., 2013; Ahmed et al., 2016; Govindpani et al., 2017; Kwakowsky et al., 2018; Yeung et al., 2021).

This editorial summarizes the contributions to the Frontiers Research Topic “*Neuronal network dysfunction in neurodegenerative disorders*.” The objective of this Research Topic was to bring together a cross-section of studies on the advances in human and non-human research focusing on deciphering the mechanisms of neuronal network dysfunction and exploring the use of new therapeutics with selected neuronal target specificity in neurodegenerative diseases.

For this Research Topic, we received a large number of submissions from which 10 articles by a total of 54 authors from 8 countries were published. The articles included 7 original research papers along with two systematic reviews and one multi-scale computational modeling study. These articles range from brain imaging studies performed on patients (Wei et al.; Zheng et al.) to studies exploring post-mortem anatomical changes (Mazumder et al.) and using *in vitro* human (Peppercorn et al.) and animal models to explore the role of neuronal networks in neurodegenerative diseases (Xie et al.) and critical review of a proposed therapeutic target (Wood et al.) and a suggested new model of disease mechanisms in PD (Muddapu et al.).

The selective loss of neurons and other mechanisms, such as abnormal expression and function of receptors and transporters, and mutations that affect their activity lead to dysfunctional network activity in neurodegenerative diseases. Importantly, the consequences of these alterations on neuronal network activity on behavior/cognition and/or motor function are not yet well-understood (Palop et al., 2006; Govindpani et al., 2017; Yeung et al., 2021; Elbasiouny, 2022). The underlying mechanisms of selective neuronal and regional vulnerability have been difficult to dissect (Surmeier et al., 2017; Giguere et al., 2018; Wang et al., 2020). Recent developments in whole-genome technologies, *in vitro* and *in vivo* disease models, and the extensive examination of anatomical, electrophysiological, and biochemical properties of vulnerable cell populations are beginning to elucidate these basic characteristics of neurodegenerative diseases. The collection of articles in this Research Topic showcases an array of tools applied to examine neuronal network dysfunction such as diffusion tensor imaging (DTI) (Huang et al.), functional and structural magnetic resonance imaging (MRI) (Wei et al.; Zheng et al.), multi-scale computational modeling (Muddapu et al.), optogenetics (Xie et al.), deep brain stimulation and post-mortem human brain analysis (Mazumder et al.), iPSc technology (Peppercorn et al.), RNA sequencing, and Sequential Window Acquisition of All Theoretical Fragment Ion Spectra-Mass Spectrometry (SWATH-MS) (Peppercorn et al.).

With respect to network dysfunction in Parkinson's Disease (PD), Muddapu et al. developed a computational model of cortico-basal ganglia networks that are strongly implicated in levodopa-induced dyskinesia (LID). The model also considers the scale at the level of oxidative stress and calcium-driven excitotoxicity. Collectively, their model proposes that neuronal cell loss in the substantia nigra pars compacta is affected differently at the terminals compared to the soma. Brain imaging techniques have also provided more insight into clinical PD. Wei et al. specifically looked at network connectivity in different presentations of PD. Deep brain stimulation (DBS) of the subthalamic nucleus (STN) has demonstrable success in improving motor symptoms in PD. The study by Zheng et al. helps to explain the neurological bases of clinical differences between the two motor subtypes of PD. Using optogenetic strategies, Xie et al. demonstrate that STN manipulation exerts behavioral effects at the level of locomotion and abnormal involuntary movements in 6-OHDA treated rats. Mazumder et al. provide evidence that DBS in humans does not exert its therapeutic benefit through minimizing neuronal cell loss, changes in astroglial cell numbers, or levels of alpha-synuclein. These studies work to highlight the circuitry involved in DBS.

Secreted amyloid precursor protein alpha (sAPPα) is a cleavage product of APP and is involved in modulating learning and memory. Previous studies proposed that it has the potential as a therapy for preventing, delaying, or even reversing Alzheimer's disease (AD). Peppercorn et al. identified a set of genes affected by sAPPα, which will aid further investigation into the mechanism of action of this neuroprotective protein. The excitatory amino acid transporter 2 (EAAT2) also appears to be a promising therapeutic target for AD. EAAT2 is the main glutamatergic transporter in the human brain and is a key regulator of normal glutamatergic metabolism and maintenance of the excitatory/inhibitory balance and network function. The systematic review by Wood et al. demonstrates that while the findings implicate EAAT2 expressional and functional alterations as key processes in AD progression, they also highlight the need for further studies to characterize EAAT2's involvement in normal physiology and disease in human tissue and to identify compounds that can act as EAAT2 neuromodulators.

Cheng et al. explored the pathophysiological mechanism of freezing of gait (FOG) in multiple system atrophy (MSA) using RS-fMRI and found that FOG is associated with centrality of the impaired thalamus network. The examination of behavioral associations and thalamocortical connectivity suggests that the non-motor circuit plays a compensatory role in MSA.

By retrospectively analyzing DTI parameters Huang et al. report that different neuroanatomical structures are affected differently by idiopathic normal pressure hydrocephalus (INPH) related to their positions in neuronal networks and characteristics, which affects the presentation of clinical symptoms and the prognosis of shunt surgery.

A systematic review by Yap et al. on spinocerebellar ataxia type 3 (SCA3) highlights the importance of evaluating the progressive changes regarding functional connectivity of the cerebellar-cerebral neuronal networks in patients. Importantly, the clinical data show that rather than individual brain regions, the connectivity between different brain regions in distributed networks may be responsible for motor and neurocognitive function in SCA3.

Recently the focus has shifted toward therapeutics which target multiple aspects of neurodegeneration to ensure future treatment success, and the targeting of specific neuronal networks offers many potential therapeutic targets (Brichta et al., 2013; Cao et al., 2018; Stoker and Barker, 2020; Ferguson et al., 2022; Mead et al., 2022). Drug designs have also shifted from treating neurodegenerative diseases at later stages of disease progression to focusing on preventive strategies at early stages of disease development (Cao et al., 2018; Stoker and Barker, 2020; Ferguson et al., 2022). Targeting neuronal dysfunction at early stages of these disorders might offer novel and more effective therapeutic options, and understanding the neuronal network dysfunction in neurodegenerative disorders will open new insights into the fundamental processes of brain circuit function and remodeling to help uncover the etiology of these intractable neurological disorders.

In conclusion, this collection of reviews and original research for this Research Topic provides new perspectives on neuronal network changes and insights toward the value of tools which can be applied to studying neurodegeneration.

## Author contributions

All authors listed have made a substantial, direct, and intellectual contribution to the work and approved it for publication.
